# The Effects of Six-Month L-Thyroxine Treatment on Cognitive Functions and Event-Related Brain Potentials in Children with Subclinical Hypothyroidism

**DOI:** 10.4274/jcrpe.1684

**Published:** 2015-06-03

**Authors:** Özlem Sangün, Serpil Demirci, Nihal Dündar, Özgür Pirgon, Tuğba Koca, Melike Doğan, Bumin Dündar

**Affiliations:** 1 Başkent University Faculty of Medicine, Department of Pediatric Endocrinology, Adana, Turkey; 2 Suleyman Demirel University Faculty of Medicine, Department of Neurology, Isparta, Turkey; 3 Katip Çelebi University Faculty of Medicine, Department of Pediatric Neurology, İzmir, Turkey; 4 Süleyman Demirel University Faculty of Medicine, Department of Pediatric Endocrinology, Isparta, Turkey; 5 Katip Çelebi University Faculty of Medicine, Department of Pediatric Endocrinology, İzmir, Turkey

**Keywords:** Subclinical hypothyroidism, event-related potentials, children, cognitive functions

## Abstract

**Objective:**

The aim of this study was to investigate the cognitive status of children with subclinical hypothyroidism (SH) before and after L-thyroxine (L-T4) treatment using event-related potentials (ERPs) and neuropsychological tests.

**Methods:**

This prospective study was conducted on a series of 20 children with mild SH (free T4 normal and thyroid-stimulating hormone level within 5-10 µIU/L) who underwent clinical and cognitive assessment before L-T4 treatment and 6 months afterwards. The recordings of ERPs were done at the time of diagnosis and after 6 months of euthyroid state. Neuropsychiatric tests for attention, perception, close and remote memory were performed on all patients and on the control group which consisted of 20 healthy children of normal intelligence.

**Results:**

While pretreatment verbal memory (VM) and verbal recall (VR) scores of the SH group were significantly lower than those of the control group (p=0.004 and 0.012, respectively), no significant differences between the post-treatment and control groups were found in these scores after 6 months of L-T4 treatment. Post-treatment VM and VR scores were significantly higher than the pretreatment scores in the SH group (p=0.008 and p=0.0001). There were no significant differences between the pre-and post-treatment values of electrophysiological evaluation in N1, P2, P3 latencies or P3 amplitude (p>0.05), although there was a significant decrease in N2 latency in the post-treatment group (p=0.03).

**Conclusion:**

SH affects cognition in children and L-T4 replacement therapy leads to normalization of cognitive functions. Neuropsychological tests can be used as complementary measures in the evaluation of children with SH. Determining the association between ERPs and SH would contribute to the comprehensive evaluation of these children.

## INTRODUCTION

Thyroid hormones are essential for brain development starting from intrauterine life. Their deficiency is associated with peripheral and central nervous system dysfunctions (1,2). Several studies showed that mental outcome greatly improved following the introduction of systematic screening for congenital hypothyroidism and initiation of treatment at an early age, enabling patients with hypothyroidism to attend school and have a normal life (3,4). Cognitive symptoms reported in hypothyroidism include lack of initiative, inability to concentrate, impaired recall and short-term memory deficit (2). Left untreated, longstanding overt hypothyroidism can lead to irreversible cognitive impairment.

Subclinical hypothyroidism (SH) is defined as a state of increased serum thyroid-stimulating hormone (TSH) levels along with circulating thyroxine (T4) within the population reference range (5). SH was reported to occur in 1.7-5.7% of children (6,7). There is growing evidence that SH may be associated with a risk of cognitive decline.

Event-related potentials (ERPs) are being more and more commonly used to evaluate the cognitive functions quantitatively in different disease states (8,9). ERPs are those potentials of the electroencephalography which are evoked by the preparation of or for events and they include an early sensory evoked potential and a late (cognitive) response P3 component. Several authors have suggested that the P3 wave generated upon applying visual or auditory stimuli can be a useful tool for objectively assessing some cognitive functions such as attention and short-term memory (10,11). However, to date, there are no published studies on analysis of ERPs in children with SH. The objectives of this present study were: a) to determine the characteristics of ERPs in a series of children diagnosed with SH and compare them with those of a control group, b) to identify the changes that occur in ERP latency and amplitudes after L-T4 treatment and c) to establish the relation between ERPs and the cognitive state of children before and after L-T4 treatment.

## METHODS

### 

Twenty children diagnosed with SH were recruited from 9-13 years old children who were admitted to the Pediatric Endocrinology outpatient clinic of Süleyman Demirel University Faculty of Medicine, from May 2012 to January 2013. The diagnosis of SH was based on the findings of a normal free T4 (fT4) level along with a TSH level between 5-10 µIU/L. These levels were confirmed with a second measurement 4-6 weeks later. The control group also consisted of 20 children who presented to the clinic for minor illnesses such as common cold, conjunctivitis or routine screening. Children who had any systemic disease, including thyroiditis, diabetes mellitus, those taking medications/iodine-containing drugs and those who had a condition known to effect TSH action or thyroid hormones secretion (e.g. glucocorticoid therapy) were excluded. The study protocols were approved by the institutional review board of the University Ethics Committee. Signed informed consent and assent forms were obtained from the parents and the children.

The diagnosis of SH children was based on the medical history, clinical findings and serum levels for free triiodothyronine (fT3), fT4 and TSH. Serum levels of fT3, fT4, TSH, antithyroglobulin antibody and antithyroid peroxidase antibody were measured using the electrochemiluminescence immunoassay method by Beckman Coulter DxI 800 analyser (Beckman Coulter Access kit, Beckman Coulter, Inc. • 250 S. Kraemer Blvd. • Brea, CA 92821 USA). The normal reference ranges for fT3, fT4 and TSH applied in our study were 1.4-4.2 pg/mL, 0.69-1.2 ng/dL and 0.5-4.7 mIU/L, respectively.

The patients came from middle class families. Intelligence was not evaluated in this study, but all participants were reported to show a normal academic performance at school. Each participant took part in two sessions that included the recording of ERPs and laboratory investigations. Following the first session, L-T4 treatment was started at a dose of 1 µg/kg/day and the dose was titrated individually every 4-6 weeks until normal TSH values were achieved. The second session was conducted 6 months after maintaining the euthyroid state.

### Auditory P3

The P3 wave was evoked by auditory oddball paradigm (12). We recorded auditory potentials after infrequent high-pitched (2000 Hz, 80 dB nHL, target stimulus) and frequent low-pitched (1000 Hz, 70 dB nHL, non-target stimulus) tone bursts of 0.1 ms duration (with a 20-ms plateau and a 9.9-ms rise/fall time). The subjects were seated upright and their visual attention was fixed at a marked point on the wall in front of them. They had to be cautious to the tone presented to both ears through headphones in a random sequence with a 20% probability of target stimulus (total stimuli, 200 tone bursts). During the test, each subject was asked to count only the target tones and to report the number at the end of each run. Data were recorded from 16 electrodes attached to the scalp according to the 10-20 International System and referred to linked ears. The response to the frequent tone consisted of negative (N1) or positive (P2) deflections; the response to the infrequent tone was negative (N1), positive (P2), negative (N2) or positive (P3) deflections. N1 and P2 latencies were identified in response to frequent tones and N2 and P3 in response to rare tones. Amplitude was measured as peak to peak.

It is reported that the P3 amplitude is related to the updating of working memory content, although its latency is related to the speed of stimulus evaluation. Abnormally prolonged P3 latencies were demonstrated in dementia, depression and Alzheimer’s disease (13,14,15,16,17).

The N2 component is usually observed between 200 and 400 ms after the stimulus. This ERP should be used for measuring various aspects of executive function, including go/no-go task. The N2 component is also related to the unexpectedness of the stimulus (18). The amplitude of the N2 has been reported to vary as a function of conflict and the need for cognitive control (19).

N1 wave is reported to reflect a discrimination process that is applied to the attended location (20).

### Neuropsychological Tests

Neuropsychological tests for attention, perception, close and remote (short and long-term) memory were performed by evaluating visual memory, visual spatial relationship, verbal memory (VM), verbal recall (VR), digit symbol test and digit symbol error.

Each neuropsychological test and ERPs were performed double-blindly by the same experienced neurologist (MD) and interpreted by another neurologist (SD). The room was designed to provide a silent environment where the patient could focus on the test.

For the VM test, a short story was read aloud by the interviewer (neurologist) putting special emphasis on certain words and the subjects were asked to remember those words. The VM score is identified as the number of words which were remembered.

For the VR test, a list of 15 words were read aloud to the subjects and the number of the words which they remember was counted. This procedure is repeated for three times and the mean value is recorded as test score.

The digit symbol test was performed in 90 seconds during which the subjects were asked to write the symbols and numbers under a column. The test score was evaluated after 90 seconds and the total number the subjects were able to write was accepted as the test score.

### Statistical Analysis

The statistical analyses were performed with SPSS for Windows, version 15 (SPSS, Chicago, IL, USA). Mean or median (min-max) values were used appropriately, as descriptive statistics. Differences in the means of variables were tested using both parametric and nonparametric tests depending on the distribution of the variables. Differences between groups were tested using the Mann-Whitney U test or the student’s t-test, as appropriate. The correlations among numerical data were analyzed by the Spearman’s coefficient (r). Pre- and post-treatment values were tested by using Wilcoxon test. A p-value of less than 0.05 was considered statistically significant.

## RESULTS

The characteristics of the study population are shown in Table 1. Gender and age of the subjects were similar in the SH and the control groups. Although within normal ranges in both groups, mean pretreatment fT4 level of the study group was lower than that of the control group (p=0.035). Mean pretreatment fT3 levels were similar between the two groups. Hashimoto’s thyroiditis was detected in 9 of the 20 patients. Four of these patients were positive for one antibody and 5 were positive for both anti-thyroid antibodies. Mean VM and VR scores of the SH group were significantly lower than those of the control group before L-T4 treatment (p=0.004 and 0.012, respectively), while visual memory and visual spatial relation scores were similar in the two groups.

As expected, pretreatment TSH values in the SH group were significantly higher than those of the control group. In the SH subjects, an euthyroid state was reached with a median L-T4 dose of 0.83 μg/kg/d (0.5-1-5 μg/kg/d) in a median duration of 47 days (31-64 days). At the 6th month of treatment, mean fT3, fT4 and TSH levels were 3.36±0.47 pg/mL, 0.86±0.14 ng/dL and 2.58±1.37 mIU/L, respectively. There was no statistically significant difference between the fT4 values of the control and post-treatment groups.

Visual memory, VM and VR scores significantly improved after L-T4 treatment (p=0.008, 0.001 and 0.006, respectively). Results of the pre- and post-treatment neuropsychological tests are shown in Table 2. The present study revealed significant shortening of N2 in SH patients after 6 months of euthyroid state as compared to pretreatment values (p=0.03) (Table 3). No significant changes in the latencies of N1, P2 and P3, nor in the amplitude of P3 waves were found. Spearman’s rho correlation was applied to see the correlation between the various waves of ERPs and TSH, fT3 and fT4 values. There was no significant correlation between the TSH values and ERPs or neuropsychiatric tests.

## DISCUSSION

There is a complete gap of consensus in the field of SH. It has long been debated whether this condition is a normal variation of thyroid function and if the L-T4 replacement has any beneficial effect. There are some population-based studies in adults which have investigated the effect of SH on quality of life, signs and symptoms and cognitive function; however, all the reported results were controversial (21,22,23,24,25,26,27,28). The effects of SH and its treatment on cognitive development is of particular interest in children, since it is well known that hypothyroidism in young children has effects on brain development. There exist some inconsistent data in older children on an association between SH and impaired neuropsychological development.

It has been reported that “Despite being within the normal range, high TSH concentrations are associated with a lower cognitive function and low fT4 with attention deficit hyperactivity disorder symptoms in healthy preschoolers. Statistically significant differences were observed in the highest quartiles of TSH” (29). Although this study makes us think that SH may influence attention and other cognitive functions negatively, another study reported that the mean reading and block design scores in SH children were higher than those of euthyroid subjects (6). It was hypothesized that children with SH were more successful because of the decreased activity and lower arousal levels associated with the SH state. However, it was noteworthy that the cognitive assessment scores of these SH patients were much lower than the standardized data among healthy adolescents. Although, Wu et al (6) stated that this situation might be related with the suboptimal conditions of the test environment, the cognitive tests were applied to all subjects under the same conditions. Therefore, the assessment scores for the different groups would indicate the relative levels of cognitive function among the groups and deserve consideration (6). As a limitation of the study, the authors declared that the number of adolescents with SH was rather small (22 in 1327 adolescents). In this present study, the number of SH patients was similar to that in the aforementioned study, but the neuropsychological tests were performed by an experienced neurologist in a room which was specially designed for this purpose. The results of our double-blind study indicate that VM scores in SH subjects were significantly lower than those of the control group.

Aijaz et al (30) followed 11 children who had thyroid hormone replacement for an average period of 3 months and no differences in neuropsychological test scores were found before and after treatment. We suggest that the inconsistency between the results of the present study and those of Aijaz et al (30) could be due to the relatively larger sample size and the longer follow-up period of our study group. Although the sample size of our study group was relatively small, we believe that this kind of longitudinal studies contribute much to the debate on the possible medical and psychiatric consequences of SH.

Törel Ergür et al (31) investigated the neuropsychological effects of SH on 17 Turkish children and reported that the children in the SH group, as compared to a control group, yielded significantly lower scores on both the Digit Span subtest and the subtests, which are sensitive to attention.

In the present study, the visual memory, VM and VR scores of the SH patients were significantly lower than those of the control group before treatment. However, no difference in test scores was noted in the evaluation between patients and the control group after six months of L-T4 treatment. These results can be interpreted to indicate that SH leads to some deterioration of cognitive functions and moreover, that treatment with L-T4 causes normalization of these scores. In a study on adults, a slight but significant improvement of verbal fluency was also obtained after 6 months of treatment with L-T4 in a group of patients with SH (32).

Verbal encoding appears to be left-lateralized in the medial temporal lobe, but its functional neuroanatomy can vary (33). The posterior parietal cortex manipulates mental images, while the visual cortex receives information from the visual field and subcortical regions. Locations are preferentially processed in ventral (occipito-temporal) and dorsal (occipito-parietal) cortical visual streams (34). Visual memory, VM and VR scores significantly improved after the L-T4 treatment in our SH subjects. We interpreted these results to indicate that, for reasons that are not clear, the brain areas mentioned above were more significantly affected. Human memory is composed of many connected parts. Visual and VM are very active parts of cognitive systems and it may be that they are more sensitive to the SH state because of their high energy demand.

By using a digit n-back paradigm and a functional magnetic resonance imaging, Zhu et al (35) showed that SH patients scored significantly lower in the 2-back task than the euthyroid subjects and that their working memory was impaired especially in frontal executive function. They also reported that the load effect of blood oxygen level response (which is observed in healthy subjects) in the frontal areas was recovered after 6 months of L-T4 treatment in these patients. Yin et al (36) showed that VM and spatial working memory of SH patients is impaired with abnormal activity in bilateral frontal areas and right posterior parietal lobe. They also reported that L-T4 replacement therapy can improve the memory impairment and reverse the altered neural activity network (36). In another adult study, mild hypothyroidism is reported to be related with decreased cerebral blood flow in regions mediating attention, memory and visuospatial processing (37). According to these data, the mechanism of the SH interference with cognition can be related with its effects on metabolism and on oxygenation of specific parts of the brain. Our results which also showed the impairment of VM and visual memory in SH are consistent with these conclusions. Significant improvements noted in the neuropsychiatric test scores in addition to the decreased latency of N2 wave after 6 months of L-T4 treatment are considered as further evidence of cognitive decline associated with SH.

ERPs are widely accepted as an objective tool for evaluating cognitive function. Khedr et al (38) found a decrease in amplitude and a significant prolongation of P3 latency in 26% of hypothyroid patients and suggested that neurocognitive function was affected in hypothyroidism. Ozata et al (39) found that adult hypothyroid patients had longer P3 and N1 wave latencies as compared to controls and reported these findings reverted to control values 6 months after attaining euthyroidism. Relationships have been established in patients with Alzheimer’s disease, Parkinson’s disease, multiple sclerosis and post-traumatic syndrome between P3 onset latency and the patient’s level of cognitive functioning (40,41,42). In our study, the latencies of the N2 in SH patients significantly decreased after 6 months of L-T4 treatment, although no statistically significant difference was observed for P3 wave.

Many different components of the ERPs have been identified including Nd, P165, NA, N1, P2, N2, P3, P3a, P3b, P4 and N (18,43). The N2 component of the ERP is usually observed at medial-frontal sites between 200 and 400 ms following stimulus presentation on various measures of executive function (44,45,46,47,48,49). Executive function refers to the deliberate, overall neurocognitive processes involved in the regulation of thought, action and emotion and it is believed to be a better predictor of achievement than IQ (50,51). In an adult study, Nazliel et al (52) reported that N2 and P3 latencies in a hypothyroid group were prolonged relative to controls and that the differences were statistically significant. Although our study group was in the pediatric age group and did not have overt hypothyroidism, we observed that the N2 component of ERPs was significantly improved after 6 months of euthyroid state and we concluded that L-T4 therapy in SH has beneficial effects on executive function which means better learning skills. Thus, this electrophysiological study showed that an optimal amount of thyroid hormone is required for normal sensory and cognitive processing.

The ERPs in children with SH and their relationship with clinical and cognitive status before and after substitution of thyroid hormone were also evaluated in this study. There was no significant correlation between thyroid hormone levels and ERPs before and after L-T4 treatment. This lack of correlation may be associated with the TSH level of our SH group, a level which was only slightly increased above normal. Further studies with larger groups are required in this field. Among the limitations of our study, we should mention the sample size, because, despite being the most extensive series in the literature, the number of our patients was also low.

In conclusion, the results of our study indicate that children with SH had mild cognitive impairment, identified by the prolongation of a latency in ERPs and lower scores in visual and VM in neuropsychological tests. The more important point is the improvement in these areas following treatment with L-T4, which is an evidence of efficacy of treatment. This study also shows that some alterations can develop in the central nervous system functions in SH patients who have a TSH value between 5-10 µIU/L. Therefore, neuropsychological tests should be considered as complementary measures in assessing the functional status of children with SH. An increase in visual and VM scores besides the decrease in N2 latency can be interpreted as the benefits of the L-T4 treatment in these patients.

## Figures and Tables

**Table 1:**
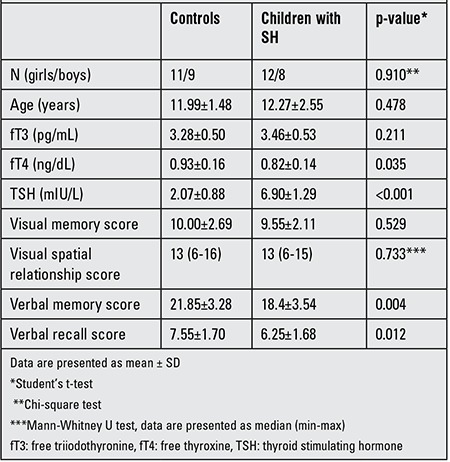
Characteristics of subclinical hypothyroidism (SH) patients at diagnosis and of controls.

**Table 2:**
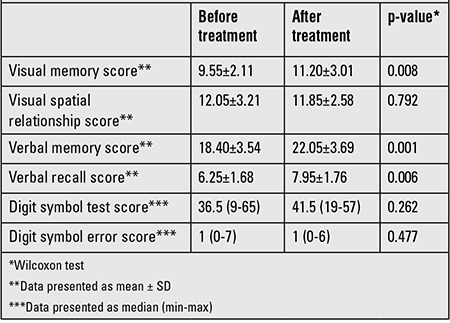
Neuropsychological test scores of the subclinical hypothyroidism patients before and after treatment.

**Table 3:**
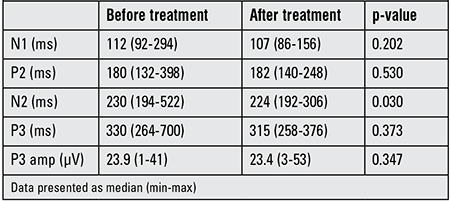
Mean latencies of event-related potentials (ERPs) and the peak amplitude of P3.
